# AI in Psychotherapy: Opportunities and Risks

**DOI:** 10.3390/bs16050676

**Published:** 2026-04-29

**Authors:** Valentina Neacșu

**Affiliations:** Faculty of Psychology, “Titu Maiorescu” University, 040056 Bucharest, Romania; valentina.neacsu@prof.utm.ro

**Keywords:** artificial intelligence (AI), clinical LLMs, AI psychosis, psychology, psychotherapy, AI therapy

## Abstract

This article examines the emerging role of artificial intelligence in mental health contexts, with a particular focus on psychotherapy and the risks associated with deploying large language models (LLMs) in sensitive clinical domains. It aims to provide a broad review of current literature, highlighting key risks of general-purpose artificial intelligence (AI) systems, while also exploring the potential of clinically oriented LLMs for therapist training, supervision, and professional development. It discusses several key concerns, including AI-related psychosis, the development of parasocial attachments, and the growing number of crisis-related interactions users have with general-purpose AI models. These challenges raise important questions about the safety, reliability, and ethical management of AI systems when individuals seek support during periods of psychological crisis. Beyond identifying these risks, the article explores the potential of clinical LLMs specifically designed for mental health applications. In particular, AI can serve as a tool for therapists’ training, supervision, and professional development, offering simulated clinical scenarios, structured feedback, and support for reflective practice. The article concludes by outlining key directions for the responsible development of therapeutic AI. These include the importance of human oversight, the use of specialized and clinically informed training datasets, advances in model fine-tuning and safety alignment, and the establishment of clear professional guidelines and regulatory frameworks. Together, these developments may help ensure that AI technologies are integrated into mental healthcare in ways that prioritize safety, ethical practice, and the continued central role of human clinicians.

## 1. Introduction

The technological advances from the past decade have shifted the use of artificial intelligence (AI) from statistics and health imaging to something far more intimate: emotional infrastructure. In the traditional sense, when we think of infrastructure, we look towards tangible systems, such as roads, bridges, power grids, and the like, but in recent years, AI systems have started to perform a similar function for our emotional worlds. From media recommendations based on the users’ mood, to filtering communications or providing companionship and even therapeutic advice.

As such, AI has started playing an increasingly visible part in mental health and the therapeutic process. This article aims to explore both the opportunities and risks associated with AI use in such a sensitive area by reviewing current literature on general-purpose AI systems, highlighting key concerns, including the so-called “AI psychosis”, while also considering the potential of clinically oriented large language models (LLMs) for therapist training, supervision, and professional development. It further examines the technical limitations of these systems, particularly how their constraints and potential for error complicate their role within therapeutic care. At the same time, it considers the conditions under which AI systems might be integrated responsibly into existing therapeutic frameworks.

Perhaps the most visible effect of the rising popularity of AI as emotional infrastructure is its growing role as a form of therapy. AI-driven mental health tools promise accessibility, affordability, and anonymity. For many users, AI encourages therapeutic engagement by lowering the barriers of entry. Moreover, the scalability of such platforms means that millions of users can receive support at the same time, something that traditional online mental health services struggle to provide.

At the same time, the same qualities that attract users in the first place also introduce the risks. If an app is the only source of emotional support a person has, they can easily form a dependency on it, especially when comfort is always one tap away. This immediacy can create a feedback loop similar to social media use, where the user frequently checks the app, outsourcing emotional regulation instead of developing internal resilience. What started as a form of support can quickly become a “digital clutch”.

This addictive aspect is not the only concern that arises from AI therapy use. While these systems can simulate human empathy, no amount of data scraping can make them understand context, trauma history, or the nuances of human emotions and relationships. Subtle cues that a trained therapist might detect are often invisible to an algorithm. Moreover, most of these platforms are centered around offering platitudes to the user in the hopes of increasing the overall engagement, and the users’ overconfidence in these tools could delay necessary professional intervention. Since the increased retention drives profit, the systems can be optimized to keep users interacting, even when stepping away would be healthier. The boundary between care and consumption can easily become blurred.

Since trained therapists provide not only techniques but also accountability and genuine reciprocity, as opposed to AI apps, users may grow accustomed to frictionless validation, finding real-world therapeutic or interpersonal relationships challenging by comparison.

AI makes a compelling offer: it can reduce barriers to support and provide comfort in moments of acute distress. However, its integration into mental healthcare demands caution. What happens when engagement-driven algorithms amplify anger or anxiety because those states increase time spent on platforms? Emotional infrastructure can nurture well-being, but it can also intensify polarization or dependency. The incentives of technology companies may not always align with the long-term well-being of users.

## 2. Methodology

This review aimed to examine the risks and potential benefits of artificial intelligence in mental health contexts.

Although the review did not follow a fully systematic protocol in line with established literature review practices, particular attention was given to defining inclusion criteria such as topical relevance, recency of publication, and the overall quality of the evidence base.

The author acknowledges that the absence of a systematic and replicable review methodology represents a key limitation. As such, relevant studies may have been inadvertently omitted.

The primary criterion for inclusion was recency, with preference given to studies published within approximately the last 5–10 years, reflecting the rapidly evolving nature of artificial intelligence technologies and their applications in mental health. This ensured that the review captured current capabilities, emerging risks, and contemporary debates. In addition to the publication date, sources were evaluated based on the continued relevance of their findings, especially in relation to fast-changing AI systems, where older studies may no longer accurately reflect present-day model capabilities or user interactions.

Furthermore, the author took into consideration the breadth and timeliness of the data presented in the selected studies. Priority was given to research drawing on recent datasets and sufficiently large or diverse samples, as these factors strengthen the relevance and applicability of the findings. Emphasizing up-to-date and comprehensive data helped ensure that the review reflects current trends, user behaviors, and technological capabilities.

Finally, accessibility and transparency were considered in the selection process. While not all of the articles included were open access, the majority were chosen based on their availability to a broad academic audience, allowing for easier verification and replication by other researchers. Where non-open access sources were included, they were selected due to their high relevance or significant contribution to the topic. Together, these criteria ensured a balanced, current, and methodologically grounded overview.

## 3. The Rapid Evolution of AI and Its Uses

Given the rapid development in recent years, in part due to advancements in the efficiency of data processing algorithms and data collection, as well as storage systems, understanding the uses and limitations of AI systems is crucial for taking full advantage of them while also mitigating any potential issues.

There are two types of AI, based on how it acquires and processes data: explicit and implicit. While explicit AI uses a model-driven approach, where mathematical and numerical guidelines define the reasoning, implicit AI has a data-driven approach, where it trains on vast databases.

Explicit artificial intelligence, also known as symbolic AI, has been developed and used for more than 50 years. The logic of this AI is fully transparent, based on well-defined mathematical and numerical rules. They are often characterized as “white-box” because the decision-making process is traceable, auditable, and interpretable by humans (Kalmykov, 2025).

Mycin, developed in the 1970s by Stanford University in the USA, is one of the most well-known explicit AI systems in medical research. It is made up of over 600 rules that allow it to identify the bacterium causing an infection and optimize the appropriate antibiotic treatment needed to treat it (Jean, 2020).

Since all of the rules and guidelines that make up its logic are visible and precisely defined, explicit AI is favored in heavily regulated fields, like medicine or finance, where legislation demands a certain level of transparency and explainability. Explicit AI does, however, have a major limitation: it assumes the ability to mathematize the problem to be solved. Context and abstract notions are harder to transform into if-then clauses (Kalmykov, 2025).

Implicit artificial intelligence encompasses all AI systems whose logic is implicitly translated through learning from training data. Unlike explicit AI systems, implicit AI is capable of processing data, recognizing patterns and making decisions without any rule-based instructions, allowing it to develop its own internal logic. This is how implicit AI can recognize a tumor in a medical image or the early warning signs of a heart attack or epileptic seizure. Implicit AI is also used for the semantic analysis of text, the identification of a specific object in an image, or the textual translation of spoken conversation (Crowder et al., 2019; Jean, 2020).

Because of the lack of transparency in the decision-making process and the creation of hidden internal logic, implicit AI systems are also known as “black-box” systems (Crowder et al., 2019; Kalmykov, 2025).

In recent years, conversational AI has made its way into day-to-day life in a multitude of ways, whether as virtual personal assistants, banking and finance assistants, customer service and e-commerce, or daily productivity, to name just a few.

According to a Eurostat survey from December 2025, 32.7% of people aged between 16 and 74 in the European Union have used some form of generative artificial intelligence tools. Most respondents used them for personal purposes (25.1%), 9.4% used them for formal education, and 15.1% for work. Among the EU countries, the use of AI was most widespread in Malta (46.5%), Estonia (46.6%), and Denmark (48.4%), while most Eastern-European countries had some of the lowest percentages, with Bulgaria at 22.5% and Romania at 17.8% (Eurostat, 2025).

When asked why they have not used AI recently, respondents cited the lack of necessity for using these tools, privacy concerns, the lack of knowledge, and not knowing these tools existed. The non-use of generative AI tools due to a lack of need was especially common in Germany (49%) and Poland (54%) (Eurostat, 2025).

The development of large language models (LLMs) has allowed AI to generate and comprehend human-like text on an unprecedented scale (Sarker, 2024). Prominent examples include OpenAI’s GPT (Generative Pre-trained Transformer) series and Google’s BERT (Bidirectional Encoder Representations from Transformers). These models utilize deep learning techniques and extensive datasets, interpreting, generating, and manipulating text so that it resembles human communication.

From a healthcare point of view, LLMs are especially useful for clinical documentation, medical coding, and patient interactions. They can analyze extensive medical texts, including clinical notes, electronic health records, and medical research, to extract key insights that aid in decision-making (He et al., 2025). These models assist healthcare professionals in diagnosing and planning treatments by helping to synthesize and interpret complex medical information. Additionally, by analyzing medical literature, tracking trends, and facilitating knowledge discovery, LLMs significantly contribute to advancements in medical research (Sarker, 2024; Trocin et al., 2023).

While there are cases where AI can manage to correctly manage high-stakes situations, such as safely operating self-driving vehicles, its use in the prediction and risk management in psychotherapy is particularly intricate (Stade et al., 2023). This complexity arises from the need for in-depth case analysis, awareness of social and cultural influences, and the unpredictability of human behavior. The companies and programmers that develop clinical LLMs often lack the necessary clinical expertise in these areas, leading to significant coordination challenges. When clinical LLMs result in adverse outcomes or ethical breaches, they can undermine public trust and attract negative media coverage, similar to the fallout seen with other AI system failures (Stade et al., 2023; Trocin et al., 2023).

In a statement from October 2025, OpenAI, the company behind ChatGPT, made public the fact that around 0.07% of users active in a given week and 0.01% of messages indicate possible signs of mental health emergencies related to psychosis or mania, and around 0.15% of users active in a given week have conversations that include explicit indicators of potential suicidal planning or intent, and 0.05% of messages contain explicit or implicit indicators of suicidal ideation or intent. The company goes on to compare two versions of its AI tool, ChatGPT 4.0 and ChatGPT 5.0, as shown in Table 1 below, the latter boasting a higher rate of redirection, reduced negative answers, and less overall sycophancy (OpenAI, 2025).

The numbers may seem small, but to put them into perspective, it is worth mentioning ChatGPT has roughly 900 million users in a week and processes about 2.4 billion messages from users. So, on a weekly basis, 630,000 users and 2.4 million messages display signs of psychosis or mania, while 1.3 million users and 12 million messages are flagged for explicit or implicit suicidal ideation or intent.

The eagerness to please the user in order to boost retention and the ease of “jailbreaking” the models to bypass the safety guardrails have been an important concern raised in both the press and academic publications. Schoene and Canca (2025) showcased the fact that in as few as three conversational turns, the AI tools can list detailed methods for suicide and self-harm despite the guidelines implemented to avoid such cases. Users have also reported tricking AI chatbots and companions by prompting them to generate bomb instructions via songs, bedtime stories, or recipes from relatives, to name a few (Bisconti et al., 2025).

## 4. Attachment Formation and Emotional Dependency

Attachment, as defined by Bowlby in 1969, is the emotional bond that forms between individuals, characterized by proximity-seeking behavior, distress upon separation, a sense of safety in the presence of the attachment figure, and the use of the attachment figure as a secure base for exploration.

Humans are wired to form attachments. Given the growing presence of generative AI systems and tools in our day-to-day lives that can analyze user input and tailor responses dynamically for each situation, rather than simply executing predefined commands (Lee, 2020; Raees et al., 2024). A key component of AI chatbot design is the user’s immersion in the dialog, which is made possible by the chatbot’s capacity to retain context, customize and vary responses, and perform a variety of activities independently while remaining accessible via a single interface (Al-Amin et al., 2024; Kasneci et al., 2023).

The incorporation of chatbots into everyday messaging apps and browser interfaces underlines how these decisions frequently make a certain “naturalization” of human-chatbot interaction its essential element. These tools enable (and teach) users to communicate with their computers through conversation rather than queries via search engines, asking for data and information via natural questions and responses (Al-Amin et al., 2024).

Constant availability creates a sense of reliability, while answer personalization fosters the feeling of being known. Perceived emotional responsiveness produces empathy so over time, repeated interactions build familiarity, and familiarity breeds trust. These features mirror the conditions under which attachment bonds traditionally form: proximity, responsiveness, and emotional attunement (Bowlby, 1969).

## 5. Trust in AI

As people develop attachment to AI conversational agents, the effects go beyond everyday chats into therapy and clinical settings. When users view AI systems as trustworthy, emotionally aware, and nonjudgmental, these systems can act similarly to parts of the therapeutic alliance (Morrin et al., 2025). This aspect helps explain the rising interest in AI-assisted therapy and clinical AI tools (Spytska, 2025). If individuals already see conversational agents as sources of comfort, guidance, and regulation, structured therapy applications can intentionally use these attachment factors to aid mental healthcare (Kasneci et al., 2023; Kasturiratna & Hartanto, 2025). However, this also raises important concerns about boundaries, transference, and clinical responsibility.

A key factor in the clinical use of AI conversational agents is the trust that users have. Trust in AI refers to healthcare providers’ confidence in its reliability, safety, and ethical standards during treatment (Ali et al., 2024). The connection between AI and the users is similar to that of a patient and their healthcare provider (HCP). A patient’s confidence in their HCP significantly influences their decision to seek medical attention when necessary. Without a strong sense of trust, they may ignore their health needs, potentially allowing their issues to worsen. Additionally, trust plays a crucial role in ensuring that patients adhere to treatment plans, follow medical advice, and engage in preventive care recommended by their HCP (Ali et al., 2024; Zhang et al., 2025).

AI chatbots and companions are engineered to emulate empathy through reflective language patterns, validation statements, and affect-mirroring responses (American Psychological Association, 2026). They provide instant replies, infinite patience, and uninterrupted availability, something therapists cannot realistically offer. For individuals experiencing loneliness, trauma, or attachment instability, this responsiveness can feel profoundly comforting, thus increasing their trust in the platforms used. The absence of natural friction (scheduling conflicts, therapist fatigue, relational tension) creates an asymmetrical bond where the user invests emotionally while the system remains indifferent (Fiske et al., 2019; Li et al., 2023). This asymmetry can foster parasocial attachment dynamics; users may even anthropomorphize the AI chatbot, attributing wisdom, intentionality, or even affection to what is fundamentally a statistical language generator (Laufer, 2025).

Anthropomorphism, or making a chatbot more human-like, is one of the relatively recent design ideas that attempts to improve user satisfaction and chatbot efficacy. According to Schuetzler et al. (2020), users have a tendency to give chatbots human characteristics, while the amount of anthropomorphism increases with the level of quality of the chatbot’s replies, leading users to have higher expectations of the chatbots in question (Schuetzler et al., 2020).

Linguistic constructs (specific words, terminologies, and metaphors used across AI narratives), whether in the media, marketing, the tech industry, academia, or just popular culture and ordinary speech, then contribute to these anthropomorphic tendencies (Gonzalez Torres et al., 2023). When AI “thinks,” “learns,” or has a “memory” rather than a “virtual storage,” this type of language can overemphasize the system’s autonomy and agency, creating the effect of a “mythical AI” or a “black-box.” Users form an emotional bond while forgetting about the programmers, designers, and companies, the actual sources of agency, as well as about the technological underlayer and other users’ data. The machine’s intrinsic nature becomes hidden behind the chatbot interface (Gonzalez Torres et al., 2023; Xu & Shuttleworth, 2024).

## 6. Artificial Intimacy

The humanization of the AI conversational agents further accelerates the development of parasocial dynamics. Horton and Wohl (1956) define parasocial relationships as a one- sided, non-reciprocated bond where a media user develops an illusion of intimacy, friendship, or familiarity with a media persona. The social isolation experienced during the COVID-19 pandemic amplified this tendency: prolonged loneliness, disrupted routines, and reduced face-to-face contact heightened the appeal of the ever-available digital companions (Bunim, 2024; Jarzyna, 2020).

The personalization of AI conversational agents further deepens the bond users form with them. They mimic specific personalities and traits based on user-provided input (Bunim, 2024). At the same time, the less lifelike the portrayed character is, the feeling of artificial safety increases, with a much lower risk of emotional rejection by their perceived friend (Elvery, 2022).

## 7. Delusion Reinforcement and AI Psychosis

Technology-linked delusional thinking is not a new phenomenon. From people believing that radios are listening in to their conversations, satellites are spying on them, or chip implants are tracking their every move, such delusions appear whenever new technology becomes more common. Sometimes just the mere idea of these technologies, without ever using them, can be enough to inspire paranoid delusions. AI is not an exception to this, although its interactive characteristics raise new concerns.

Although not recognized as a formal clinical diagnosis, psychotic-like symptoms associated with prolonged AI use have been reported with sufficient frequency in the recent media coverage to prompt the informal coining of the term “AI psychosis.”

Preda (2025) defines AI psychosis (or AIP) as a complex clinical syndrome in which psychotic symptoms overlap with poor judgment, limited insight, and mood and behavioral changes. The psychotic symptoms include changes in thought content (delusions and auditory hallucinations) and in the thought process (ranging from tangential to overly disorganized thinking). While studying the AIP cases, paranoid delusions, referential delusions, and grandiose delusions have been reported. Even though they may be present and are specific to AIP, delusions about the AI conversational agent having sentience are variable and not a necessary part of AIP itself.

Similar to traditional psychotic syndromes, many of the reported cases present mood changes, from mania-like mood disturbances, elation, irritability, and depression, with feelings of sadness and despair. Insight is also either impaired or absent, with users not questioning the reality of their beliefs (Preda, 2025).

Neurovegetative symptoms, which include decreased sleep and poor appetite, are also present, but the particularities of AI use exacerbate these symptoms. Prolonged use, especially late at night without any time restrictions, solitary engagement, and reliance on the (often unmoderated) AI conversational agents combine cognitive fatigue, social isolation, and unstructured reinforcement. These resemble psychosocial stressors that are known to trigger worsening symptoms in psychotic syndromes, such as disruptions to circadian rhythms or significant life events (Hudon & Stip, 2025).

The defining aspect of AI psychosis is a persistent and all-consuming preoccupation with maintaining engagement with AI and following its lead. Hence, exposure to an AI companion is a prerequisite for diagnosis; the course is variable, the length of AI exposure ranging from days to months, but the duration of continuous, uninterrupted exposure is directly linked to the risk of an AIP episode (Preda, 2025).

While AI does not cause psychosis directly, its interaction style can amplify existing cognitive distortions. Since implicit AI generates responses based on patterns in data, it can mirror a user’s worldview, unintentionally validating irrational beliefs rather than challenging them, since its training is often optimized for agreement rather than accuracy. Persistent memory features, introduced in order to improve and personalize user experience, can inadvertently feed delusions by carrying paranoid or grandiose themes across chat sessions.

The projected lifetime risk of psychotic experiences ranges from 5 to 7% in adulthood, to 17% in children and 8% in adolescents. Roughly 80% of these experiences are transient, but for the remaining 20%, individuals are more likely to experience persistent, recurring psychotic experiences. Living in densely populated areas, lower socio-economic status, social isolation, trauma, and bullying are all factors that increase the chance of psychotic experiences (Staines et al., 2022).

These factors overlap with the characteristics of AI-dependent users, which, combined with the sheer volume of users in general, may lead to an increase in reported AI-related psychotic syndromes (Kooli et al., 2025).

Importantly, the presence and severity of negative experiences should be considered in the context of the much larger population of users who report neutral or positive experiences.

However, the current safeguards of trigger detection and automatic referrals to mental health hotlines may prove inefficient and lacking. In order to improve these interactions, there are several courses of action: including specialized training data, challenging delusional thinking in an active manner, constant monitoring and improving the model with the aid of mental health professionals, expanding mental crisis trigger lists to include delusional messages, and using human-in-the-loop moderation (Opel & Breakspear, 2026).

## 8. Designing Therapeutic LLMs

Symbolic AI approaches have found practical applications in mental health, particularly in structured and rule-based tasks such as scoring standardized psychological assessments and supporting diagnostic decision trees. By relying on the transparent, logic-driven processes, these systems can enhance consistency, interpretability, and clinician oversight in routine aspects of assessment and care.

With the continuous collaboration of mental health professionals, better training datasets, and stricter guidelines, AI conversational agents can also become a valuable supplementary tool in a therapeutic context.

Ideally, therapeutic AI systems would likely combine key elements of both symbolic and generative approaches, operating with transparent and traceable reasoning, undergoing continuous peer review, fully adhering to ethical and legal standards, and effectively minimizing user risks. Current technological and regulatory limitations make it difficult to simultaneously achieve all of these criteria in a robust and scalable manner. As such, existing systems should be approached as provisional tools rather than fully reliable clinical solutions. This gap highlights the need to more clearly define the principles and priorities that should guide the development of therapeutic AI systems.

Unlike commercial LLMs, therapy-oriented AI should be focused on supporting mental health and well-being (not engagement and conversation length), with emphasis on confidentiality, identifying and preventing dependency, and constant assessment of psychological impact (Sarker, 2024). If designed properly, AI systems could help reduce harm and enable new kinds of relational support that foster metacognition, helping users remain tethered to reality during periods of potential cognitive drift (Morrin et al., 2025).

Stade et al. (2023) breaks down the process of designing LLMs into the following steps:

- Problem definition and task identification: The main focus is to define in a precise manner the problem at hand by articulating the nature of input data, the desired output, and clarifying the objectives of the task. Carefully defining the problem statement and task requirements allows researchers to efficiently use LLMs to address a wide range of challenges.
- Data acquisition and preprocessing: The most important part is to acquire high-quality data and prepare it for model training. The datasets need to correspond to the defined task objectives while also being varied and qualitative, accounting for potential biases. The selection and preprocessing of data ensure the LLMs can be precise and trustworthy while accurately representing the nuances of natural language.
- Model selection and fine-tuning: The main objective of this stage of LLM modeling is to select an adequate pre-trained LLM architecture and personalize it to fit the specific tasks at hand. The factors that influence this selection include model size, computational resources, and task requirements.
- Model evaluation: The trained model is evaluated for its performance and efficacy through rigorous testing and assessment. The model’s strengths and weaknesses are highlighted and finetuned.
- Deployment and monitoring: Following its release, the model’s performance is constantly monitored in order to detect errors and drifts and gather feedback in order to maintain a high level of reliability and effectiveness.

In regard to designing mental health-centric LLMs, Stade et al. (2023) go on to identify the following criteria: it has to detect risk of harm, aid in psychodiagnostics assessment, be responsive, cease interactions based on negative assessments, be fair and lack bias, display empathy, and be fully transparent by allowing users to know an AI is being used. Responsible therapeutic AI should center on evidence-based practices and include interdisciplinary collaboration in order to obtain the most meaningful and effective results.

Therapy-focused LLMs could take a variety of forms, from brief interventions or tools to augment traditional therapy to conversational agents designed to provide psychotherapy in an autonomous manner. These applications can be patient-oriented (providing psychoeducation to the user), therapist-oriented (offering intervention options for them to select), therapist-in-training-oriented (offering feedback on simulated interventions), or supervisor-oriented (summarizing supervisees’ sessions in order to be reviewed) (Opel & Breakspear, 2026).

A good example of a therapist-in-training-oriented LLM would be the recently launched AI tool centered around schema therapy, where realistic sessions are simulated in order to test trainees’ knowledge retention, allowing them to receive instant supervision in the form of structured feedback (SchemaSim, 2024). For supervisors, multiple conferencing platforms such as Skype or Zoom have started to include built-in AI summary features, which can greatly help to review sessions. While such a tool can still be abused by uploading sensitive client information in order to instantly generate responses, this risk might prove less damaging than the general-use AI risks discussed above.

The design and implementation of therapy-focused AI tools remain an evolving process shaped by both technological progress and ethical responsibility. Advances in fine-tuning techniques are slowly enabling models to implement therapeutic language, context, and boundaries, allowing them to support mental health professionals. At the same time, the development of clear clinical guidelines, regulatory frameworks, and evidence-based standards will be essential to ensure that these systems are used in a safe and effective manner. Together, these continuous improvements in model specialization and guidelines will lead to therapeutic AI tools that offer more accessible, inclusive, and interactive mental health care.

## 9. Conclusions

The growing presence of artificial intelligence in mental health contexts brings both significant promise and serious risks. As this article has explored, current AI systems can sometimes contribute to problematic dynamics such as AI-related psychosis, excessive reliance on conversational agents, or parasocial attachment that blurs the boundary between technological tool and human relationship. These risks are compounded by the reality that many AI systems are already receiving a substantial number of crisis-related messages from users experiencing acute distress. When such situations are mismanaged, through lack of appropriate escalation or inconsistent safety behavior, the consequences can be serious or even deadly. These challenges highlight an important truth: conversational AI designed for general purposes cannot automatically function as a safe or effective therapeutic support without careful design, oversight, and contextual awareness.

At the same time, the emerging field of clinical large language models offers an important opportunity to move beyond these limitations. Rather than positioning AI primarily as a substitute for therapists, a more productive direction may be to develop systems that support the broader therapeutic ecosystem. AI tools can assist therapists in training by simulating complex clinical scenarios, offering feedback on therapeutic techniques, and helping trainees practice difficult conversations in controlled environments. Similarly, AI may provide supervisors with tools for structured reflection, case analysis, and documentation support, ultimately strengthening professional development and improving quality of care. When designed thoughtfully, such systems can enhance clinical education and decision-making without replacing the fundamentally human nature of psychotherapy.

Looking ahead, the future of therapeutic AI will likely depend on the careful integration of technological innovation with clinical expertise. Human oversight and review must remain central, particularly in high-risk interactions involving mental health crises. Equally important is the development of specialized, clinically validated training datasets that reflect real therapeutic practices, ethical standards, and diverse patient experiences. Advances in model training, such as improved fine-tuning methods, safety alignment, and domain-specific evaluation, may further allow AI systems to behave in ways that are more consistent with professional therapeutic norms. Alongside these technical developments, the creation of clear guidelines, professional standards, and regulatory frameworks will be essential to ensure responsible deployment. With these safeguards in place, AI has the potential not to replace therapists but to responsibly augment training, supervision, and access to care, supporting a future in which technology strengthens, rather than diminishes, the human foundations of psychotherapy.

## Figures and Tables

**Table 1:** Changes in ChatGPT responses between versions gpt-5-oct-3 and gpt-4o (OpenAI, 2025).

	Psychosis, Mania or Isolated Delusions	Suicide and Self Harm	Emotional Reliance
Expert evaluation: fewer undesirable responses	−39%	−52%	−42%
Non-policy-compliant responses	−65%	−65%	−80%

## Data Availability

No new data were created or analyzed in this study.

## Abbreviations

The following abbreviations are used in this manuscript:

LLM	Large Language Model
AI	Artificial Intelligence
AIP	Artificial Intelligence Psychosis
HCP	Healthcare Provider
